# Meningovascular Neurosyphilis Presenting as Multifocal Stroke: A Case Report and Literature Review

**DOI:** 10.7759/cureus.102049

**Published:** 2026-01-22

**Authors:** Usamah Al-Anbagi, Abdulqadir J Nashwan, Harris Poolakundan, Hatem A Abdulmajeed, Mohamed G Mohamedali, Imran H Mohammed

**Affiliations:** 1 Department of Internal Medicine, Hamad Medical Corporation, Doha, QAT; 2 Department of Nursing and Midwifery Research, Hamad Medical Corporation, Doha, QAT; 3 Department of Clinical Imaging, Hamad Medical Corporation, Doha, QAT

**Keywords:** meningovascular neurosyphilis, mri brain, penicillin g therapy, rehabilitation, stroke mimic, treponema pallidum

## Abstract

Neurosyphilis (NS) remains a diagnostic challenge due to its diverse clinical manifestations and its ability to mimic other neurological disorders. Despite the global decline in syphilis incidence during the antibiotic era, a recent resurgence has led to an increase in atypical and meningovascular presentations. Early diagnosis is critical, as timely treatment can prevent irreversible neurological damage. We report the case of a 38-year-old immunocompetent male who presented with acute vertigo, unsteady gait, and subsequent confusion. Initial imaging and laboratory investigations were unremarkable; however, MRI revealed multifocal ischemic lesions. Serological testing demonstrated high-titer *Treponema pallidum* antibodies (rapid plasma reagin 1:256), while CSF analysis showed lymphocytic pleocytosis, elevated protein, and an increased IgG index, with a negative CSF VDRL. The diagnosis of meningovascular NS (MVS) was established. The patient received intravenous penicillin G for three weeks, resulting in gradual cognitive and functional recovery. After rehabilitation, his Functional Independence Measure score improved from 83 to 121, and his Mini-Mental State Examination score improved from 9 to 21. MVS should be considered in patients presenting with stroke-like symptoms, particularly when conventional vascular risk factors are absent. A negative CSF VDRL does not exclude the diagnosis; combined clinical, serologic, and radiologic evidence is essential. Early recognition and appropriate antibiotic therapy can lead to significant neurological recovery.

## Introduction

Syphilis, caused by *Treponema pallidum*, is known as the “great imitator” due to its protean clinical manifestations and its ability to affect multiple organ systems, including the CNS [1]. Neurosyphilis (NS) refers to CNS involvement that may occur at any stage of infection, manifesting as asymptomatic, meningitic, meningovascular, or parenchymatous forms [2,3]. Although once rare, NS has reemerged globally in parallel with the rising incidence of syphilis, a trend driven by changing sexual practices, reduced early screening, and delayed treatment [1,4]. This resurgence is increasingly observed among younger adults and immunocompetent patients, reflecting a broader shift in transmission and disease patterns [1,4].

Meningovascular NS (MVS) is a distinct subtype characterized by chronic inflammatory endarteritis, leading to cerebral infarction due to vascular occlusion [1,5]. It accounts for 10-15% of all NS cases and typically presents years after primary infection [1,3]. The middle cerebral and basilar arteries are most frequently affected, although small-vessel vasculitis may also occur [5]. Clinically, MVS may mimic ischemic stroke, autoimmune vasculitis, or demyelinating disorders, often delaying appropriate diagnosis [1,6].

Diagnosis remains challenging because the classical CSF VDRL test, although highly specific, has limited sensitivity and may be negative even in confirmed cases [1,2]. Therefore, integration of clinical findings, MRI changes, and positive serum treponemal and nontreponemal tests is critical for accurate diagnosis [3]. MRI often reveals multifocal ischemic lesions or diffuse white-matter changes, while angiography may demonstrate concentric stenosis of cerebral arteries [1,6].

Intravenous penicillin G remains the treatment of choice for NS, with documented improvement in neurological outcomes when administered early [1,4]. Here, we present a case of MVS in an immunocompetent male initially misdiagnosed as ischemic stroke, highlighting diagnostic pitfalls and reviewing current evidence from recent literature.

## Case presentation

History

A 38-year-old male, recently diagnosed with thyrotoxicosis secondary to Graves’ disease, presented to the emergency department with a five-day history of vertigo. The vertigo was sudden in onset, nonpositional, and not associated with nausea or vomiting. He described a sensation of the surroundings spinning, accompanied by an unsteady gait and a tendency to sway to the right. The patient also reported generalized weakness and fatigue since symptom onset. He denied headache, visual blurring, diplopia, tinnitus, hearing loss, ear pain, or ear discharge. There was no history of recent upper respiratory tract infection, trauma, fever, or seizures. He did not report tremor, restlessness, excessive sweating, numbness, paresthesia, confusion, or memory impairment. Additionally, there were no symptoms suggestive of cranial nerve deficits, motor weakness, sensory loss, or autonomic dysfunction.

Of note, the patient reported a persistent, pruritic, generalized rash that progressively worsened over the past month, without mucosal involvement or systemic allergic manifestations. His medications included carbimazole 5 mg twice daily and propranolol 80 mg once daily. The patient is married and denied any other sexual relationships. He had no history of thromboembolic events, connective tissue disease, or other autoimmune disorders. He was a nonsmoker and a teetotaler and reported no relevant family history of thyroid or neurological disorders.

Examination

On examination, the patient appeared alert but mildly drowsy, with a Glasgow Coma Scale (GCS) score of 15/15, indicating spontaneous eye opening, full orientation, and obeying commands. He was oriented to person but not to place or time, and he answered simple questions appropriately. Neck stiffness was absent, and Kernig’s and Brudzinski’s signs were negative.

**Figure 1:**
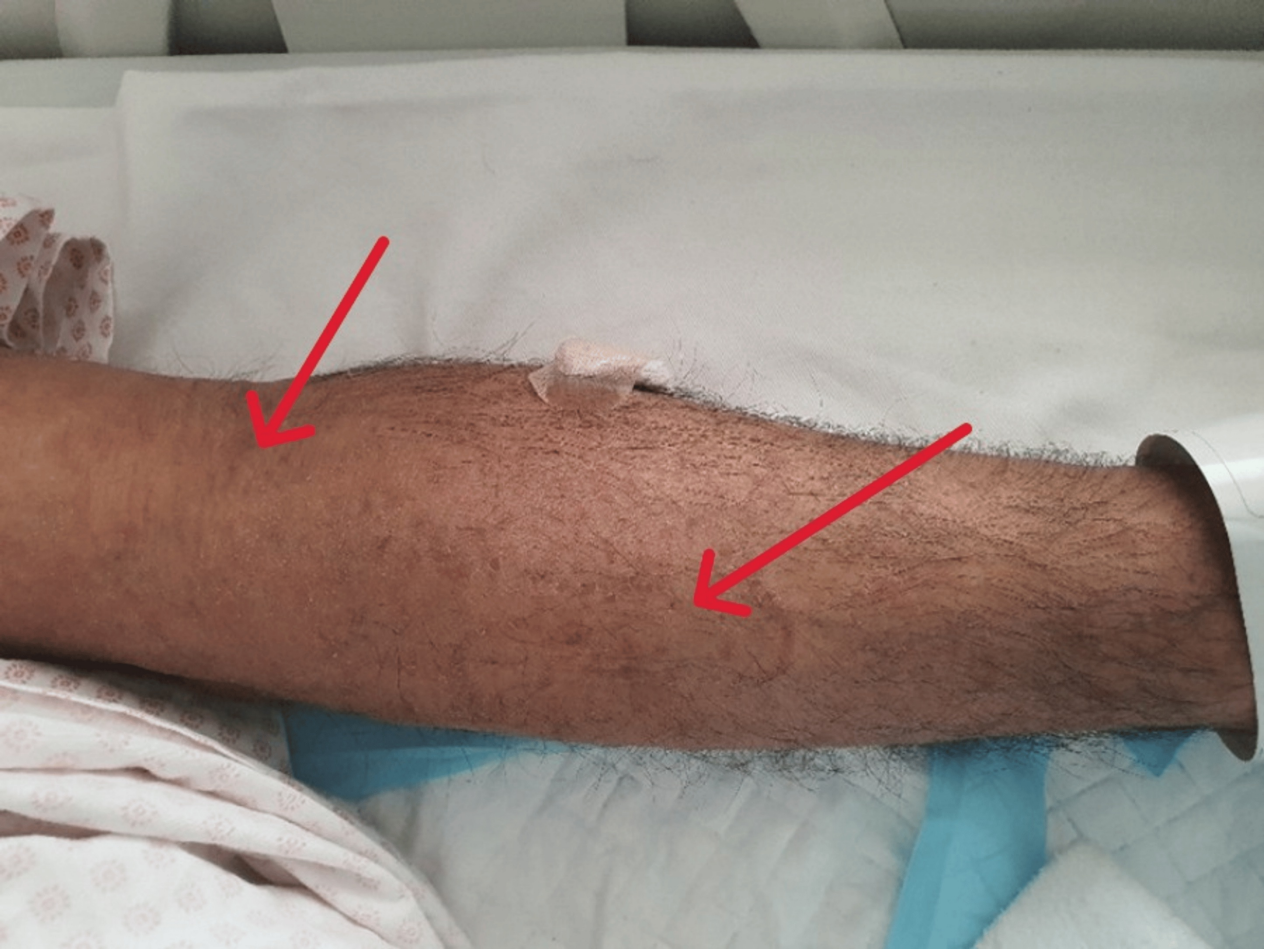
Multiple faint erythematous to brownish maculopapular lesions over the upper limb (red arrow)

**Figure 2:**
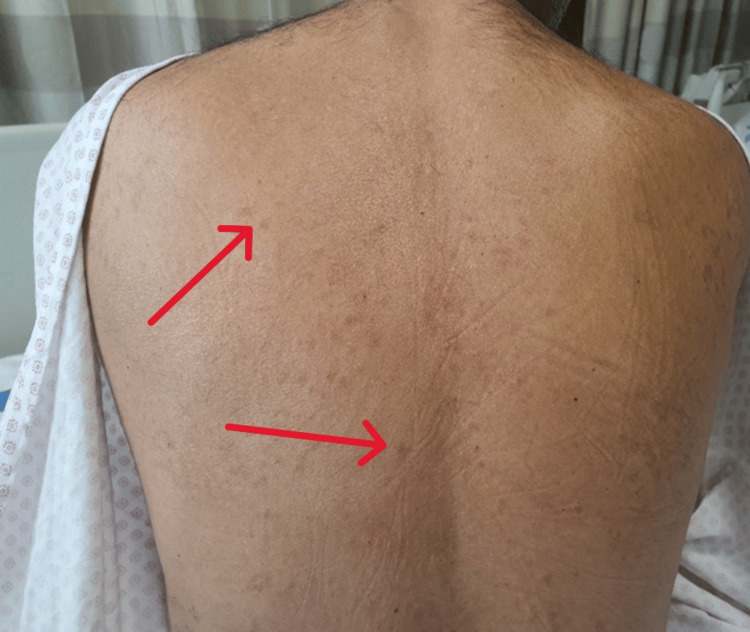
Symmetric faint maculopapular rash over the trunk with mild hyperpigmentation

Vital signs were stable: temperature 36.6 °C, heart rate 60 bpm, respiratory rate 15 breaths/min, blood pressure 140/78 mmHg, and oxygen saturation 99% on room air. There was no pallor, jaundice, cyanosis, clubbing, edema, or lymphadenopathy. A generalized maculopapular rash was noted over the body, including the palms and forearms (Figure 1, Figure 2), and a diffuse goiter was observed.

Neurological examination revealed a conscious but confused patient, disoriented to place and time, with no focal neurological deficits or meningeal signs. Cranial nerves, motor function, sensory function, reflexes, coordination, and gait were all within normal limits.

Cardiovascular examination showed normal jugular venous pressure and regular heart sounds (S1 and S2), without murmurs or other abnormal sounds. Respiratory examination demonstrated equal bilateral air entry with normal vesicular breath sounds and no adventitious sounds. Abdominal examination revealed a soft, nontender, nondistended abdomen with no palpable organomegaly.

Management and outcome

Initial laboratory investigations, including complete blood count, renal and liver function tests, and C-reactive protein, were all within normal limits (Table 1).

**Table 1:** Laboratory results ALT, alanine aminotransferase; APTT, activated partial thromboplastin time; anti-AMA-M2, anti-mitochondrial M2 antibody; anti-centromere B, anti-centromere B antibody; anti-dsDNA, anti-double stranded DNA; anti-histones, anti-histone antibody; anti-JO1, anti-histidyl tRNA synthetase antibody; anti-nucleosomes, anti-nucleosome antibody; anti-PCNA, anti-proliferating cell nuclear antigen antibody; anti-PM-Scl, anti-polymyositis scleroderma antibody; anti-RNP, anti-ribonucleoprotein antibody; anti-Ro52, anti-Ro52 antibody; anti-SS-A, anti-Sjogren’s syndrome A antibody; anti-SS-B, anti-Sjogren’s syndrome B antibody; anti-Sm, anti-Smith antibody; AST, aspartate aminotransferase; CRP, C reactive protein; FT4, free thyroxine; INR, international normalized ratio; PT, prothrombin time; TSH, thyroid stimulating hormone

Parameters	On admission	On day 7	On discharge	Reference values
Total leukocytes (× 10³/µL)	2.6	12.5	6.7	6.2
Hemoglobin (g/dL)	14	14.9	12.8	13-17
Hematocrit (%)	41.1	41.7	37.5	40-50
Platelet (× 10³/µL)	347	261	285	150-410
CRP (mg/L)	<2	9.9	<2	0-5
Serum urea (mmol/L)	2.2	6.3	5.4	2.5-7.8
Serum creatinine (µmol/L)	76	64	73	62-106
Serum sodium (mmol/L)	137	136	138	133-146
Serum potassium (mmol/L)	4.7	4.1	4.7	3.5-5.3
Serum total protein (g/L)	74	64	63	60-80
Serum albumin (g/L)	39	35	32	35-50
Alkaline phosphatase (U/L)	106	97	104	40-129
ALT (IU/L)	18	37	68	0-41
AST (IU/L)	33	81	65	0-41
Serum total bilirubin (mg/dL)	6	19	3	0-21
HbA1c (%)	6.1	-	-	<6
TSH (mIU/L)	0.74	-	-	0.34-4.20
FT4 (pmol/L)	12.1	-	-	11-23.3
PT (seconds)	11.4	11.3	9.9	9.4-12.5
INR	1	1	0.9	<1
APTT (seconds)	36.3	31.6	33.3	25.1-36.5
ANA profile (includes anti-dsDNA, anti-Ro52, anti-SS-A, anti-nucleosomes, anti-Sm, anti-RNP, anti-histones, anti-PCNA, anti-SS-B, anti-ribosomal P protein, anti-JO1, anti-AMA-M2, anti-centromere B, and anti-PM-Scl antibodies)	Negative	-	-	Negative
Factor II	Normal	-	-	Normal
Factor V	Normal	-	-	Normal
Lupus anticoagulant	Not detected	-	-	Not detected
Protein C activity (%)	120	-	-	70-140
Protein S activity (%)	92	-	-	72-126
Anti-thrombin activity (%)	83	-	-	79.4-130
Rapid plasma reagin	Reactive 1:256	-	-	Negative
Treponemal antibody	Positive	-	-	Negative
*Treponema pallidum* hemagglutination assay	Positive	-	-	Negative
HIV	Negative	-	-	Negative
PCR tests: cytomegalovirus, human herpesvirus 6, herpes simplex virus 1 and 2, varicella zoster virus, human parechovirus, enteroviruses; bacterial: *Escherichia coli *K1, *Haemophilus influenzae *type B, *Listeria monocytogenes*, *Neisseria meningitidis*, group B *Streptococcus*; fungal: *Cryptococcus neoformans*, *Cryptococcus gattii*	Negative	-	-	Negative

**Figure 3:**
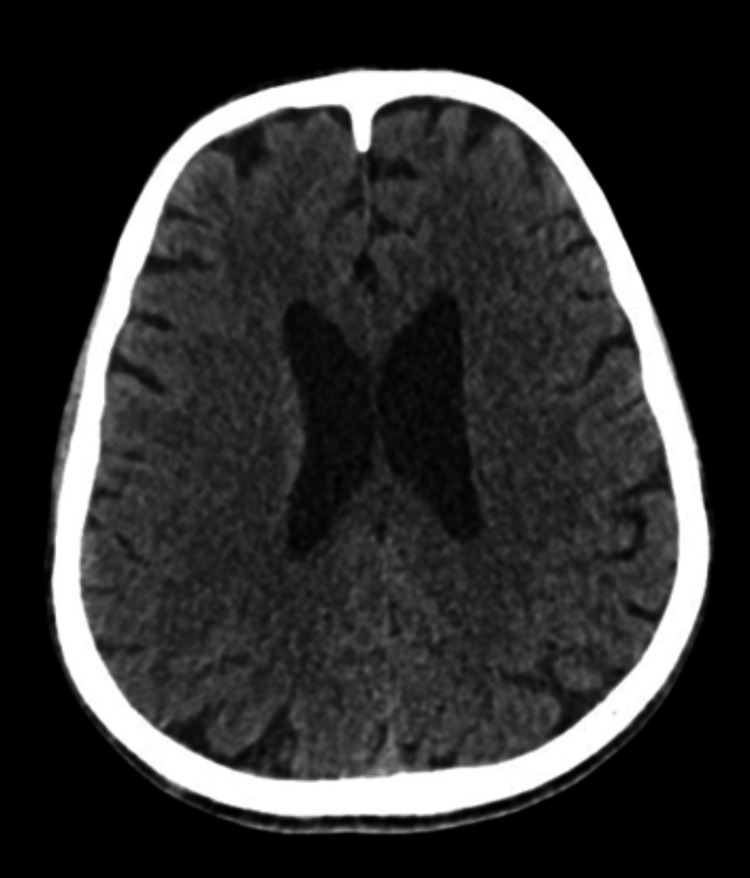
Noncontrast CT of the head (sagittal view) showing no acute abnormalities

The patient was admitted for evaluation of disorientation and dizziness, with a working diagnosis of stroke. A noncontrast CT of the brain (Figure 3) and a chest X-ray were unremarkable, as was transthoracic echocardiography.

**Figure 4:**
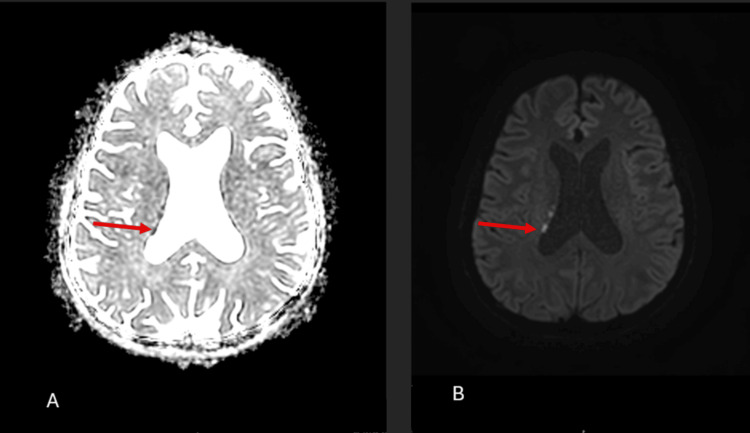
MRI of the head (A) Axial T1-weighted view. (B) Diffusion-weighted imaging view. Multiple bilateral deep cerebral parenchymal areas of restricted diffusion are noted, involving the posteromedial temporal lobes, both thalami, the posterior limbs of the internal capsules, and the deep parietal juxtaventricular regions (red arrows).

The following day, an MRI with diffusion-weighted imaging revealed multiple small foci of diffusion restriction in the right deep periventricular white matter, particularly in the peritrigonal region, suggestive of acute ischemic lesions (Figure 4).

Based on these findings, he was started on aspirin (75 mg daily), clopidogrel (75 mg daily), and atorvastatin (40 mg daily) with a working diagnosis of ischemic stroke. By the second day of admission, the patient became increasingly confused and disoriented and exhibited inappropriate behavior. Given his generalized pruritic rash, a comprehensive workup was performed. Autoimmune screening and infectious serologies were negative except for a rapid plasma reagin titer of 1:256, *T. pallidum *antibody, and treponemal hemagglutination tests, all of which were positive. Other bacterial and viral studies, including blood and urine cultures, tuberculosis screening, and viral serologies, were negative (Table 1).

A lumbar puncture revealed 36 cells/mm³ (90% lymphocytes), elevated protein, and increased CSF IgG concentration and IgG index, while CSF VDRL was negative (Table 2).

**Table 2:** CSF analysis CSF analysis demonstrated lymphocytic pleocytosis, mildly elevated protein, and increased intrathecal IgG synthesis, indicated by a high IgG index and positive oligoclonal bands. NS, neurosyphilis

Parameter	Result	Reference range/normal values	Interpretation
Color	Colorless	Colorless	Normal
Appearance	Clear	Clear	Normal
Total nucleated cells (cells/mm³)	35 ↑	0-5 cells/mm³	Elevated (pleocytosis)
Red blood cells (cells/mm³)	6	0	Slightly elevated (likely traumatic tap)
Lymphocytes (%)	96 ↑	40-80%	Lymphocytic predominance
Glucose (mmol/L)	3.39	2.2-3.9 mmol/L	Normal
Protein (g/L)	0.47 ↑	0.15-0.45 g/L	Mildly elevated
IgG (mg/dL)	51 ↑	0-34 mg/dL	Elevated
IgG index	0.7 ↑	<0.6	Elevated (suggests intrathecal IgG synthesis)
Albumin (mg/L)	237	120-350 mg/L	Normal
Oligoclonal bands	Absent	Absent	Normal
CSF VDRL	Nonreactive	Nonreactive	Negative (does not exclude NS)
CSF culture	No growth	No growth	Normal
Cryptococcal antigen	Negative	Negative	Normal

In the context of positive syphilis serology, multifocal cerebral infarcts, and compatible clinical findings, the diagnosis was revised to MVS. The patient was started on intravenous penicillin G, 4 million units every four hours, for two weeks, following infectious disease protocol. A dermatology consultation was obtained for the rash; topical fusidic acid was initiated, and a skin biopsy revealed perivascular lymphohistiocytic infiltrates in the superficial dermis, with no spirochetes identified.

**Figure 5:**
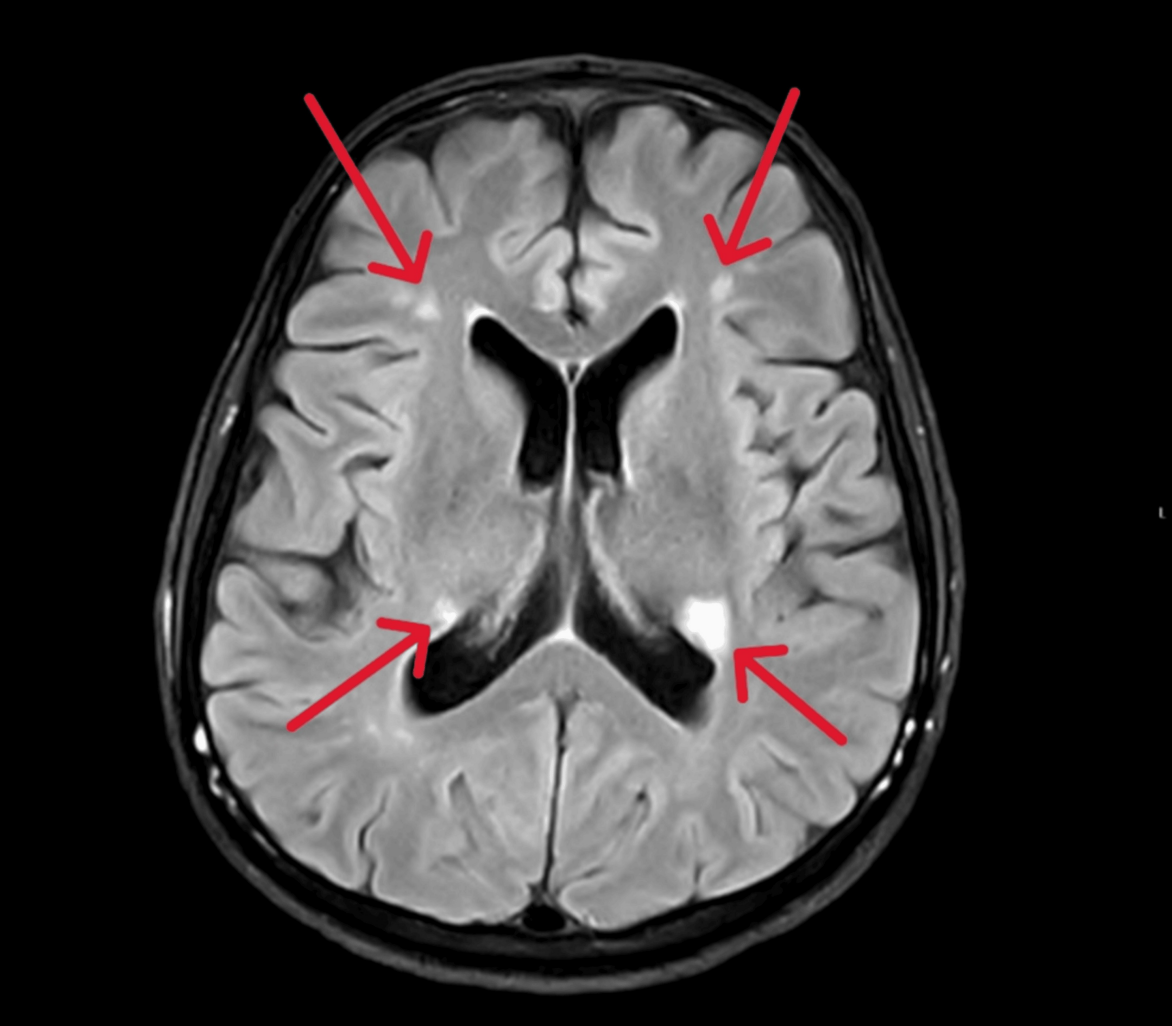
Brain MRI showing multiple bilateral deep cerebral ischemic changes (red arrows)

By day 6, the patient remained confused with ongoing delirium and had a GCS score of 12/14, though he was hemodynamically stable. A repeat MRI of the brain and spine with contrast demonstrated multiple bilateral deep cerebral ischemic changes, likely secondary to vasculitis, without abnormal leptomeningeal or spinal enhancement (Figure 5).

By day 10, his mental status had improved slightly, with a GCS of 14/15, although mild delirium persisted. He began following simple commands and participated in physiotherapy and rehabilitation. Given his gradual but incomplete recovery, the infectious disease team extended intravenous penicillin therapy to three weeks.

By the end of the third week, the patient showed some improvement in confusion and overall functional status. Laboratory parameters had normalized (Table 1), and he was transferred to the rehabilitation unit for intensive therapy. At that time, he remained partially dependent, requiring supervision for activities of daily living due to cognitive impairment and poor dynamic standing balance. His Functional Independence Measure (FIM) score was 83/126, Disability Scale 3, and Mini-Mental State Examination (MMSE) score was 9/30, indicating significant deficits in orientation, attention, recall, and constructional ability.

After approximately five weeks of inpatient rehabilitation, his neurological and cognitive functions improved markedly. At discharge, his FIM score had increased to 121/126, the Disability Scale improved to 2, and the MMSE improved to 21/30. He achieved independent ambulation, good balance, and functional speech recovery, as reflected by a Montreal Cognitive Assessment score of 25/30 and Sunnybrook facial grading of 100%. Language, communication, and fine motor coordination had normalized.

He was discharged home in stable condition, oriented, cooperative, and able to perform self-care with minimal supervision. A plan was made for ongoing outpatient rehabilitation, along with follow-up by neurology, infectious disease, and endocrinology.

## Discussion

NS continues to challenge clinicians due to its protean manifestations and the lack of a single diagnostic gold standard. Although antibiotic therapy has dramatically reduced its prevalence since the early 20th century, recent epidemiological data demonstrate a concerning global resurgence of syphilis and, consequently, NS, including atypical presentations such as meningovascular disease [3,7,8].

The incidence of syphilis has risen sharply in both high-income and developing countries, with reports of 30-40% annual increases across Europe and Asia [7,8]. NS can develop at any stage of infection, though MVS typically manifests several years after primary exposure [9,10]. Its pathogenesis involves* T. pallidum *invasion of meningeal and vascular structures, producing endarteritis obliterans, known as Heubner’s or Nissl-Alzheimer arteritis, leading to luminal narrowing, thrombosis, and focal ischemia [8]. This vasculitic process most commonly affects the middle cerebral artery and its branches but may involve deep white-matter perforators, explaining the multifocal infarcts seen in our patient [1,2].

MVS often presents with stroke-like episodes, confusion, or psychiatric changes, particularly in younger patients without conventional vascular risk factors [9,10]. Recent reviews emphasize that up to half of NS cases are initially misdiagnosed, most commonly as ischemic stroke or autoimmune encephalitis [8,11]. Psychiatric manifestations, such as delusions, hallucinations, and disorientation, occur in up to 40% of cases and may obscure the vascular etiology [12].

Diagnosis remains difficult because no single laboratory parameter confirms NS. CSF VDRL remains the most specific test, yet its sensitivity rarely exceeds 70%, particularly in MVS, where spirochete density is low [13,14]. Therefore, negative CSF VDRL results do not exclude disease. Supporting evidence includes lymphocytic pleocytosis, elevated protein, or an increased CSF IgG index [13,15]. In resource-limited settings, clinicians may initiate empiric therapy based solely on positive serology and clinical suspicion, a practice shown to yield favorable outcomes [16]. Recent systematic reviews also highlight novel diagnostic approaches, such as PCR amplification of *T. pallidum *DNA and measurement of CXCL13 chemokine in CSF, which demonstrate higher sensitivity, though these remain unavailable in most centers [13,17].

MRI plays an important adjunctive role. Diffusion-weighted and FLAIR sequences typically reveal multiple small ischemic lesions or patchy white-matter hyperintensities reflecting vasculitis [2,10]. Patel et al. emphasized the value of MRI in differentiating NS from other vasculitides and its utility in monitoring treatment response [10]. Our patient’s imaging findings, such as bilateral deep white-matter infarcts without large-artery occlusion, fit this characteristic pattern.

Intravenous aqueous penicillin G remains the standard of care for NS at a dose of 3-4 million U every four hours for 10-21 days [9,10]. In severe meningovascular cases or when the initial response is slow, treatment may be extended to three weeks, as was done in this case. For patients allergic to penicillin, ceftriaxone (2 g daily for 10-14 days) provides a reasonable alternative, though data suggest slightly lower cure rates [9,10]. Adjunctive corticosteroids are sometimes used to mitigate the Jarisch-Herxheimer reaction and inflammatory edema, although evidence remains limited [18].

Our patient demonstrated gradual neurological and cognitive recovery after a three-week course of intravenous penicillin and intensive multidisciplinary rehabilitation. The improvement in his FIM from 83 to 121 and MMSE from 9 to 21 mirrors outcomes reported in recent studies, in which 50-80% of patients show partial or complete recovery with early therapy [2,7,8]. Rehabilitation is essential to maximize neuroplasticity and functional outcomes, especially in cases with residual cognitive impairment or ataxia [1].

Recent literature underscores that NS is reemerging not only in immunocompromised populations but also among immunocompetent adults [7,8,19]. Global surveillance efforts, such as the European “No to Syphilis” initiative, stress the need for early screening and clinician awareness to prevent advanced neurological complications [7]. Lack of standardized diagnostic criteria remains a significant barrier to timely recognition. Boog et al. advocate for a unified international definition combining serologic, clinical, and CSF findings to reduce underdiagnosis [12].

## Conclusions

This case highlights several critical clinical insights. MVS can closely mimic ischemic stroke, particularly in patients lacking traditional vascular risk factors. A negative CSF VDRL result does not exclude the diagnosis when clinical presentation, serological findings, and neuroimaging are consistent with NS. Most importantly, early initiation of intravenous penicillin therapy and structured multidisciplinary rehabilitation can lead to marked neurological and functional recovery. Heightened clinical awareness and timely intervention remain essential to improving outcomes in this potentially reversible but often overlooked condition.
